# Long noncoding RNAs as regulators of pediatric acute myeloid leukemia

**DOI:** 10.1186/s40348-022-00142-2

**Published:** 2022-05-20

**Authors:** Sina Neyazi, Michelle Ng, Dirk Heckl, Jan-Henning Klusmann

**Affiliations:** 1 Department of Pediatric Hematology and Oncology, University Medical Center Hamburg-Eppendorf, Hamburg, Germany; 2 Department of Pediatrics I, Martin Luther University Halle-Wittenberg, Halle, Germany; 3 Department of Pediatrics, Goethe University Frankfurt, Frankfurt, Germany

## Abstract

Long noncoding RNAs (lncRNAs) are increasingly emerging as regulators across human development and disease, and many have been described in the context of hematopoiesis and leukemogenesis. These studies have yielded new molecular insights into the contribution of lncRNAs to AML development and revealed connections between lncRNA expression and clinical parameters in AML patients. In this mini review, we illustrate the versatile functions of lncRNAs in AML, with a focus on pediatric AML, and present examples that may serve as future therapeutic targets or predictive factors.

## Background

Acute myeloid leukemia (AML) accounts for approximately 20% of acute leukemias in children [1]. Although the overall survival of children with AML has significantly increased as a result of intensified therapy, hematopoietic stem cell transplantation, and improved supportive care over the past decades, around 25% of all patients still cannot be cured [2] — highlighting the urgent need to transfer discoveries about the molecular features of pediatric AML into new therapeutic approaches. Among the recent scientific developments in this field, comprehensive studies have revealed that the molecular landscape of childhood AML is shaped not only by oncogenic mutations and cytogenetic alterations but also by global changes in DNA methylation and gene expression affecting both protein-coding genes and noncoding RNAs [3, 4]. Noncoding RNAs in particular are emerging as important regulators of hematopoiesis and leukemogenesis and represent a largely understudied space in the search for new therapeutic strategies.

Long noncoding RNAs (lncRNAs) — defined as transcripts longer than 200 nucleotides that lack open reading frames — represent the largest group of noncoding RNAs and constitute two-thirds of the human transcriptome [5]. Different structural domains enable their interaction with RNA, DNA, and proteins and thereby allow the regulation of every stage of gene expression. Apart from their versatile roles in gene regulation on every possible transcriptional and posttranscriptional level, lncRNAs can directly interact with signaling pathways and contribute to the function of organelles such as exosomes or mitochondria.

## Molecular mechanisms and functions of lncRNAs

Based on the mechanistic interaction of lncRNAs with other molecules, four different archetypes of lncRNA functions — namely signal, decoy, guide, and scaffold — have been defined in a seminal work by Wang and Chang in 2011 [6]. As the first archetype, signal lncRNAs, which are under precise transcriptional control, act as a molecular signal reflecting a specific developmental stage, cellular background, or a response to stimuli [6–9]. LncRNAs belonging to the second archetype, decoy, bind and titrate away regulatory proteins or RNAs, thereby repressing transcription or translation of a target gene [10, 11]. Guide lncRNAs, which represent the third mechanistic archetype, direct regulatory protein complexes, chromatin modifiers, or transcription factors to their target site, resulting in either transcriptional activation or repression of the respective genomic locus [12, 13]. The fourth archetype, scaffold, describes lncRNAs as a structural platform at which different bound components of protein complexes or ribonucleoprotein complexes are assembled or can interact with each other [14, 15]. Even though increasing evidence now points toward complex lncRNA mechanisms that represent rather coexisting or overlapping features of the four classical archetypes, these four mechanistic subtypes still illustrate the wide range of possible modes of action of lncRNAs.

Nuclear-localized lncRNAs can exert independent regulatory effects on neighboring genes (function *in cis*) as well as on distant genes (function *in trans*), while cytoplasmic lncRNA mechanisms include competitive miRNA binding and interaction with translational proteins [16]. The most commonly demonstrated function of c*is*-acting nuclear lncRNAs, which are operating at their own site of transcription, is regulation of gene expression and chromatin modification [17]. The participation of *cis*-acting lncRNAs in transcriptional processes is supported by the high abundance of lncRNA genes in the proximity of regulatory elements of the human genome such as enhancers and promotors [18]. Protein-coding genes that are involved in transcriptional regulation, e.g., genes encoding transcription factors or chromatin modifiers, show a higher enrichment of closeby lncRNA genes than protein-coding genes of other functional categories, further indicating an essential contribution of *cis*-acting lncRNAs to the regulation of gene expression [19]. LncRNAs can act in *cis* to either activate or repress the expression of nearby genes through a variety of mechanisms. For instance, lncRNAs can activate gene expression in *cis* by recruiting proteins that establish spatial interactions such as chromatin loops, thereby enabling closer contact of an enhancer to the respective protein-coding gene [20, 21]. Other lncRNAs have been shown to activate gene expression of their target genes in *cis* in an transcript-independent manner by the process of their own transcription and splicing through recruiting cofactors, accumulation of transcriptional proteins, and establishing of activating chromatin marks [22, 23]. Conversely, *cis*-acting lncRNAs can also recruit chromatin modifiers that repress transcriptional activity at their genomic locus, such as the polycomb repressive complex 2 [24, 25]. Another lncRNA mechanism that results in decreased expression of the neighboring target gene is transcriptional interference: The transcription of a lncRNA can interfere with the transcription of the adjacent gene by impeding recruitment of necessary proteins such as transcription factors and chromatin remodeling proteins or by increasing nucleosome density, thereby preventing transcription factor access [26, 27]. For *trans*-acting lncRNAs, diverse functions in the modulation of distant gene expression have been demonstrated, with most of the studied examples exhibiting mechanisms that have been also described in the context of *cis*-acting lncRNAs. For instance, lncRNAs can facilitate transcriptional activation of distant target genes by the initiation of chromatin loop formation [28]. Similarly, several lncRNAs have been shown to repress transcription of target genes in *trans* through recruitment of chromatin-modifying complexes [7, 9, 29]. Another mechanism that has been described for *cis*-acting lncRNAs as well as for *trans*-acting lncRNAs is the formation of RNA-DNA hybrids, the so-called R-loops, that are recognized by transcription factors or chromatin modifiers and thereby lead to the activation or repression of transcription of the target gene [30].

Given their versatile cellular and molecular functions, it is no surprise that lncRNAs are involved in many essential physiological processes such as genomic imprinting and differentiation, as well as in the pathogenesis of diseases such as cancer, neurodegenerative disorders, and metabolomic diseases [31–33].

## LncRNAs in hematopoiesis and AML

Hematopoietic differentiation is a tightly regulated, hierarchically ordered process coordinated by the expression of specific gene programs. Numerous lncRNAs have been characterized in the context of hematopoiesis including lncRNAs that are involved in hematopoietic fate decision and lncRNAs whose deregulation contributes to the malignant transformation of hematopoietic progenitor cells [34]. Recent studies identified unique stage- and lineage-specific lncRNA signatures in distinct blood cell populations indicating an important contribution of lncRNAs to the homeostasis and regulation of hematopoiesis [3, 35, 36]. Here, we review a selection of well-characterized lncRNAs that are involved at different levels of hematopoietic differentiation.

Fetal lncRNA *H19* is one of the best-characterized lncRNAs in embryonic development and tumorigenesis. Physiologically downregulated after birth, *H19* is expressed in almost every type of human cancer [37, 38]. During embryonic development, the lncRNA facilitates the transition from endothelial cells to hematopoietic stem cells (HSCs), whereas in adult hematopoiesis, it is essential for maintaining HSC quiescence, thereby regulating the long-term homeostasis of HSCs [39, 40]. In addition, *H19* is overexpressed in AML and correlates with poor prognosis. In vitro knockdown of *H19* leads to decreased proliferation and increased apoptosis in AML cell lines — further supporting its potential oncogenic effect in AML [41]. Another example of a lncRNA implicated in HSC homeostasis is *LncHSC-2*, a nuclear lncRNA, which is expressed in HSCs and hematopoietic progenitors [42]. *LncHSC-2* regulates long-term self-renewal and lymphoid differentiation of HSCs by binding to Tcf3, a transcription factor that is essential for HSC proliferation and differentiation into myeloid-lymphoid progenitor cells [42].

In addition to these and other mechanistically studied examples of lncRNAs involved in HSC maintenance and maturation, several comprehensive transcriptomic studies identified hundreds of lncRNAs enriched in HSCs that are co-expressed with lineage-specific transcription factors, indicating that lncRNAs represent another important layer of the complex regulatory network that tunes hematopoietic differentiation [35, 36, 42]. Accordingly, lncRNAs are specifically enriched and functionally relevant not only in the context of HSCs but also in hematopoietic progenitor cell populations and mature blood cell populations. For instance, lncRNA *HOTAIRM1* is highly expressed during granulocytic differentiation and contributes to the modulation of target genes in *cis* and in *trans* that are essential for proper myelopoiesis [43].

*LINC00173* is another example of a lncRNA that is essentially involved in myeloid differentiation. We identified *LINC00173* to be specifically expressed in mature granulocytes [3]. Upon knockdown in hematopoietic stem and progenitor cells (HSPCs), granulocytic differentiation and phagocytic capacity are impaired, whereas the erythroid lineage remained unaffected. Further analyses revealed a direct interaction between *LINC00173* and PRC2, as well as differential H3K27 trimethylation at the promoter regions of genes involved in stemness, megakaryopoiesis, and erythropoiesis [3].

During erythroid differentiation, lncRNA *EPS* is enriched only in erythroid progenitor cells and promotes terminal erythrocytic differentiation by repressing pro-apoptotic pathways [44]. Within the lymphoid lineage, numerous lncRNAs that regulate differentiation and contribute to the immune response have been described. An example is lncRNA *NeST*, which is specifically expressed in CD4+ T-helper 1 cells and regulates the transcription of inflammatory genes through recruiting histone methyltransferase complexes [45, 46].

Dysregulation of the hematopoietic system results in uncontrolled proliferation of immature progenitor cells and in a block of proper differentiation, ultimately leading to the development of leukemia. Several lncRNAs have been shown to contribute to leukemogenesis [34]. In addition, lncRNAs may also serve as biomarkers or predictive factors in this disease [34]. It has been demonstrated that specific lncRNA expression profiles can be utilized to distinguish between different known molecular and cytogenetic AML subgroups and may serve as independent predictors of clinical outcome [47]. Using several examples, we illustrate different functions that have been described for individual lncRNAs in AML. We also briefly discuss lncRNA loci that may have functional consequences in AML cells independent from the encoded transcripts.

The lncRNA *HOTAIR* is highly expressed in AML and serves as a predictor for poor clinical outcome [48]. In vitro studies in primary AML blasts suggest an oncogenic function of *HOTAIR*, where it supposedly acts as a decoy for the tumor-suppressive microRNA miR-193a [48]. An alternative mode of action has also been described, where *HOTAIR* exerts its oncogenic effect in AML through EZH2-mediated epigenetic silencing of the tumor suppressor gene *p15* [49].

LncRNA *ANRIL*, which is upregulated in both AML and ALL, acts as an oncogenic lncRNA by epigenetic silencing of its antisense tumor suppressor gene *p15* [14, 50] As for other oncogenic lncRNAs, recent studies indicate a correlation of ANRIL expression to poor survival in patients with AML [51].

In contrast to the previous examples, lncRNA *IRAIN* is downregulated in AML cell lines and patients with high-risk AML, indicating that lncRNAs might not only act as oncogenes but as tumor suppressors in AML, too [52]. This lncRNA is transcribed antisense from the insulin-like growth factor type I receptor (*IGF1R*) locus, which is known to promote proliferation of AML cells through the PI3K/Akt signaling pathway [53, 54]. Mechanistically, *IRAIN* is involved in the formation of an intrachromosomal chromatin loop connecting the IGF1R promoter to a putative enhancer element [52]. However, the functional implications of this mechanism have yet to be elucidated. Clinical data further support the suggested tumor-suppressive function of *IRAIN* in AML, demonstrating a correlation between low *IRAIN* expression and poor prognosis in non-M3 acute myeloid leukemia patients [55].

While the majority of lncRNAs have yet to undergo in-depth characterization, this selection of examples provides a glimpse into the diversity of lncRNA functions in the context of hematopoiesis and AML. Even for these better-studied examples, it should be noted that mechanistic details remain elusive, due in part to the extensive experimental labor required to discern between RNA-dependent and -independent effects originating from lncRNA loci [56]. As a case in point, our group recently described *MYNRL15* — a pan-myeloid leukemia dependency locus involved in genome topology, whose lncRNA product is dispensable for its dependency phenotype [57]. We found CTCF-enriched lncRNA loci (C-LNCs) like *MYNRL15* to be enriched for leukemia vulnerabilities and provide a catalog (www.C-LNC.org) in hopes of facilitating the functional classification of lncRNAs and the discovery of new oncogenic vulnerabilities [57].

## LncRNAs in pediatric AML

In contrast to many other malignant diseases, AML occurs in all age groups, but children account for only a small proportion of all patients with AML. The molecular landscape of pediatric AML differs significantly from the molecular profile of adult AML. Chromosomal aberrations are more common in children than in adults with AML [4, 58]. In addition, the genes that are frequently mutated in adult AML (*NPM1*, *DNMT3A*, *IDH1*, *IDH2*, *RUNX1*, *TP53*) are less often affected in children. Other genes, such as *FLT3* or *GATA2*, differ in terms of the exact location and frequency of the mutations between pediatric and adult AML [4]. Given these biological and clinical differences, it is essential that we refrain from simply transferring new findings from adult cell lines, mouse models, and clinical cohorts of adult AML patients to the pediatric setting. Rather, investigations that focus specifically on pediatric AML are needed, to refine current risk stratification criteria and to develop novel therapeutic strategies for children with AML. While most current examples of lncRNAs with roles in AML have first been described in adult contexts, there is now an increasing number of studies characterizing lncRNAs in pediatric AML.

Our group described, for the first time, subtype-specific lncRNA signatures for six major cytogenetic subgroups of pediatric AML, namely, Down syndrome (DS) and non-DS acute megakaryoblastic leukemia (AMKL), inv[16], t[8;21], and AML with *KMT2A* rearrangement (t[9;11] and t[10;11]) [3]. In the transcriptional landscape of normal and malignant hematopoiesis, most DS- and non-DS-AMKL samples, and *KMT2A*-r samples, cluster in close proximity to HSCs. Their lncRNA expression profiles are characterized by the absence of myeloid expression programs. In contrast, all other pediatric AML samples clustered in proximity to normal myeloid progenitor cells. We further uncovered a core lncRNA stem cell signature that is shared between HSCs and AML blasts of all different pediatric AML subgroups. High expression of this core lncRNA stemness program is significantly correlated with poor survival in a cohort of adult AML patients [3].

Other studies have focused on the in-depth characterization of individual lncRNAs in pediatric AML. Here, we will summarize lncRNAs that have been implicated in pediatric AML (Table 1) and will exemplarily discuss several individual lncRNAs, for which molecular functions have been studied in the context of pediatric AML. Luo et al. found that the lncRNA *HOTTIP* is overexpressed in *NPM1*-mutated and *KMT2A*-r AML cases and predicts poor outcome [59]. Mechanistically, they showed that *HOTTIP* alters the three-dimensional structure of the nearby *HOXA* locus and binds to posterior *HOXA* sites as well as other genes critically involved in hematopoiesis and leukemogenesis, resulting in the activation of an AML-specific transcriptional program. Of note, *HOTTIP* expression is sufficient to initiate leukemic transformation of HSCs in mice. Knockout of *HOTTIP* perturbs leukemic proliferation and prolongs the survival in AML mouse models, suggesting a novel therapeutic option for the treatment of pediatric AML [59].

**Table 1:** LncRNAs implicated in pediatric acute myeloid leukemia

Name	Role in pediatric AML	Cellular function	Clinical significance in pediatric AML	References
HOTTIP	Oncogenic in *NPM1*-mutated and *KMT2A*-r AML	Activation of posterior *HOXA* genes and other hematopoietic genes	High expression correlates with poor survival	[59]
CDK6-AS1	Oncogenic	Silencing of *RUNX1* transcription and activation of mitochondrial biogenesis	High expression correlates with poor treatment response	[60]
UCA1	Oncogenic	Binding of various miRNAs	Unknown	[61–65]
MONC	Oncogenic in AMKL	Unknown	Unknown	[66]
MIR100HG	Oncogenic in AMKL	Unknown	Unknown	[66]
MEG3	Tumor suppressive	Activation of *p53* expression and *DNMT3A*	High expression correlates with better survival	[67–69]
HOXA10-AS	Oncogenic in *KMT2A*-r AML	Activation of the NF-κB pathway	High expression correlates with poor survival	[70]
LINC00998	Tumor suppressive	ZFP36 binding and reduction of mTORC2 mRNA stability	Low expression correlates with poor survival	[71]
LINC01257	Oncogenic in t(8;21) AML	Unknown	High expression correlates with poor survival	[72]
MVIH	Oncogenic	Unknown	High expression correlates with poor treatment response and survival	[73]
GAS6-AS1	Oncogenic	Decoy for tumor-suppressive miRNA miR-370-3p	Unknown	[74]
FBXL19-AS1	Oncogenic	Unknown	High expression correlates with poor survival	[75]
SNHG14	Oncogenic	Decoy for tumor-suppressive miRNA miR-193-3p	Unknown	[76]
DARS-AS1	Oncogenic	Decoy for tumor-suppressive miRNA miR-425	High expression correlates with poor survival	[77]
TUG1	Oncogenic	Decoy for tumor suppressive miRNA miR-221-3p	Unknown	[78]
LINC00909	Oncogenic	Decoy for tumor-suppressive miRNA miR-625	High expression correlates with poor survival	[79]
LAMP5-AS1	Oncogenic in *KMT2A*-r AML	Activation of DOT1L and global H3K79 methylation	High expression correlates with poor survival	[80]
LINC0064	Oncogenic	Decoy for tumor-suppressive miRNA miR-378a	Unknown	[81]
Lnc-SOX6-1	Oncogenic	Unknown	High expression correlates with poor survival	[82]
CCAT1	Oncogenic in t(8;21) AML	Unknown	High expression correlates with poor survival	[83]
PVT1	Oncogenic in t(8;21) AML	Unknown	High expression correlates with poor survival	[83]
CASC15	Oncogenic in t(8;21) AML	Regulation of YY1-mediated transcription of *SOX4*	No correlation to prognosis	[84]
DLEU2	Tumor suppressive in AML M5	Unknown	No correlation to prognosis	[85]

A recent elaborate study has identified lncRNA *CDK6-AS1* as a novel regulator in pediatric AML [60]. In a pediatric patient cohort, *CDK6-AS1* was significantly overexpressed and associated with higher minimal residual disease after induction therapy. High *CDK6-AS1* levels contributed to an immature phenotype in healthy HSCs and primary AML blasts, whereas silencing of the lncRNA led to increased hematopoietic differentiation of HSCs and to a rescue of the pathogenic undifferentiated state of AML blasts. Mechanistically, the authors could show that *CDK6-AS1* regulates expression of its neighboring gene *CDK6* by sharing a bidirectional promotor, and that the common *CDK6-AS1/CDK6* axis downregulates *RUNX1* signaling, which is essential for early hematopoietic differentiation. In addition, *CDK6-AS1* activates mitochondrial biogenesis in healthy HSCs as well as in pediatric AML blasts. Interestingly, mitochondrial targeting, using Tigecycline, sensitizes AML blasts with high *CDK6-AS1* expression to chemotherapy, supporting the concept of a mitochondrial vulnerability in these blasts. Overall, these findings identified *CDK6-AS1* as an important regulator of early hematopoietic differentiation and leukemogenesis of pediatric AML and uncovered therapeutics targeting mitochondrial biogenesis as a novel treatment strategy in pediatric AML [60].

*UCA1* is an additional example of an oncogenic lncRNA in adult and pediatric AML. *UCA1* has been shown to be upregulated by CEBPα-p30, the CEBPα isoform that results from CEBPA mutations recurrently found in AML patients [86]. In pediatric and adult AML cell lines, *UCA1* is upregulated, and knockdown of the lncRNA impairs leukemic viability, migration, and invasion through binding of various microRNAs, such as miR-126, miR-204, miR96-5p, and miR296-3p [61–64]. Furthermore, *UCA1* contributes to chemoresistance in pediatric AML by tethering miR-125a [65].

Other examples of relevant lncRNAs in childhood AML are *MONC* and *MIR100HG*, which are host genes for the homologous miRNA clusters miR-99a~125b-2 and miR-100~125b-1, respectively. These miRNA clusters are known to promote the progression of AMKL [87, 88]. Both lncRNA host genes are highly expressed in AMKL cells compared to cell lines of other pediatric AML subtypes. Lentiviral overexpression of *MONC* alters hematopoietic differentiation, independently of the expression of the oncogenic miRNA clusters [66].

LncRNA *MEG3* is downregulated in adult and pediatric AML, supposedly by epigenetic modifications of its genomic locus. Mechanistically, *MEG3* has been shown to activate *p53* expression and DNMT3A, thereby inhibiting leukemogenesis [67]. Hypermethylation of the *MEG3* promoter is associated with poor prognosis in adult AML patients [68]. In pediatric AML patients, higher expression of *MEG3* correlates with better survival [69].

These are only a few examples of lncRNAs that have been characterized in the context of pediatric AML and that show correlation to clinically relevant subgroups and/or prognosis of the patients. In addition, a more general lncRNA scoring system based on the expression of 14 lncRNAs has been proposed to predict overall survival in children with AML [89].

## *HOXA10-AS*: a novel oncogenic lncRNA in pediatric AML with *KMT2A* rearrangements

In a recently published study from our group, the antisense lncRNA *HOXA10-AS* was identified as an essential regulator of hematopoiesis and as a novel oncogenic lncRNA in the context of pediatric AML with *KMT2A* rearrangements (*KMT2A*-r AML) [70]. Along the genome, *HOXA10-AS* is located at the posterior end of the *HOXA* cluster — one of the four highly conserved *HOX* gene clusters. Tightly controlled spatiotemporal expression of the different *HOX* genes is crucial for hematopoietic differentiation, and dysregulation of *HOX* genes such as *HOXA9* and *HOXA7* by KMT2A fusion proteins is responsible for leukemic transformation in *KMT2A*-r AML [90]. The *HOX* gene clusters harbor numerous lncRNAs that are expressed in the same specific pattern as their protein-coding neighbors during differentiation, indicating their putative biological importance [9]. Previous studies revealed that *HOX* lncRNAs are capable of regulating the expression of neighboring or distant protein-coding *HOX* genes, as well as of independent effects on other signaling pathways [9, 12, 91]. Although a handful of *HOX* lncRNAs have undergone further characterization, the role of the vast majority of *HOX* lncRNAs in AML remains unknown.

**Fig. 1:**
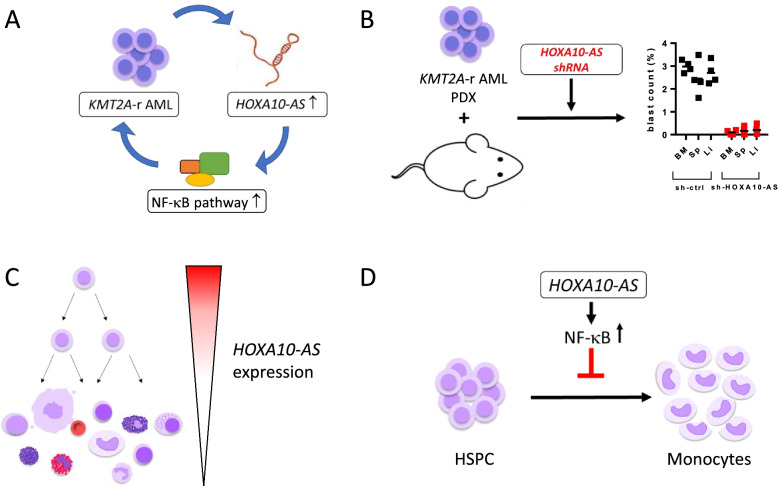
*HOXA10-AS*, an example of a lncRNA regulator of hematopoiesis and pediatric leukemia. **A** *HOXA10-AS*, is overexpressed in pediatric AML with *KMT2A* rearrangements, where it increases leukemic proliferation via activation of the NF-κB signaling pathway. **B** Knockdown of *HOXA10-AS* using shRNAs leads to reduced growth of *KMT2A*-r AML patient blasts in vivo. **C** *HOX10-AS* is specifically expressed in hematopoietic stem cells and downregulated during hematopoietic differentiation. **D** Upon overexpression in hematopoietic stem and progenitor cells (HSPCs), *HOXA10-AS* impairs monocytic differentiation through the activation of NF-κB target genes

*HOXA10-AS* is transcribed from the antisense strand relative to the protein-coding gene *HOXA10* and microRNA mir-196b, both of which are involved in hematopoiesis and in the pathogenesis of *KMT2A*-r AML. In our study, we confirmed that *HOXA10-AS* is overexpressed in *KMT2A*-r AML as well (Fig. 1A). *KMT2A* rearrangements are the most frequent cytogenetic aberrations in pediatric AML and predominantly affect infants [58, 92]. Gain-of-function experiments in cell lines and primary blasts showed increased leukemic growth of *KMT2A*-r AML cells upon *HOXA10-AS* overexpression. In complementary loss-of-function assays using shRNA-mediated knockdown, CRISPR-Cas9-induced excision, and LNA-GapmeRs, we further demonstrated that the maintenance of *KMT2A*-r AML cells depends on high *HOXA10-AS* expression (Fig. 1B). During normal hematopoiesis, *HOXA10-AS* is specifically expressed in HSCs and strongly downregulated during hematopoietic differentiation, whereas the neighboring genes *HOXA10* and mir-196b remain highly expressed in early myeloid progenitor cells (Fig. 1C). This strict stem cell-specific expression of *HOXA10-AS* suggests an independent regulatory circuit and cellular function separate from that of the nearby genes. Hematopoietic differentiation assays upon lentiviral overexpression of *HOXA10-AS* in HSPCs revealed impaired monocytic differentiation in *HOXA10-AS* overexpressing cells (Fig. 1D). The observations that ectopic expression of *HOXA10-AS* impairs monocytic differentiation and that *HOXA10-AS* is overexpressed in *KMT2A*-r AML are consistent with the fact that *KMT2A*-r AML predominantly manifests as a monoblastic leukemia (AML FAB M5) [58]. Regarding the mechanistic characterization of *HOXA10-AS*, we found that its effects in hematopoiesis and leukemogenesis were independent of changes in expression of the neighboring oncogenes, arguing against a regulatory role for *HOXA10-AS* on the *HOXA* cluster *in cis*. This was supported by subcellular localization studies, which showed that the *HOXA10-AS* is mainly located in the cytoplasm. Indeed, microarray-based gene expression analysis uncovered a possible *trans* mechanism for *HOXA10-AS* involving the upregulation of NF-κB target genes in *HOXA10-AS* expressing early monocytic progenitors and *KMT2A*-r AML (Fig. 1 A and D). Finally, we provided a proof of principle of how *HOXA10-AS* could be leveraged towards clinical implementation, by demonstrating *HOXA10-AS* as a prognostic marker in AML and potential therapeutic target in pediatric *KMT2A*-r AML [70].

## Conclusion

LncRNAs are emerging as regulators of hematopoiesis and AML pathogenesis, and knowledge about their individual effects is rapidly increasing. However, extensive functional research is required before we gain a complete understanding of the complex regulatory networks surrounding lncRNAs and their interplay with known oncogenic drivers. While individual examples continue to provide valuable information about the roles of lncRNAs and how they might serve as novel therapeutic targets or prognostic factors in AML, research on lncRNAs in pediatric AML still lags behind adult AML. Thus, investigations of lncRNAs such as *HOXA10-AS* add important insights on the regulatory roles of lncRNAs in general, as well as crucial knowledge about the specific pathogenesis of pediatric AML, both of which will hopefully contribute to a comprehensive view and new therapies for this disease.

## Data Availability

Not applicable.

## Abbreviations

AML: Acute myeloid leukemia
AMKL: Acute megakaryoblastic leukemia
DS: Down syndrome
HSC: Hematopoietic stem cell
HSPC: Hematopoietic stem and progenitor cell
*KMT2A*-r AML: Acute myeloid leukemia with *KMT2A* rearrangements
lncRNA: Long noncoding RNA
shRNA: Short hairpin RNA
